# Hemophilia’s Overlooked Female Face

**DOI:** 10.3390/jcm15062155

**Published:** 2026-03-12

**Authors:** Alkistis Adramerina, Marina Economou

**Affiliations:** 1st Pediatric Department, Faculty of Medicine, School of Health Sciences, Aristotle University of Thessaloniki, Hippokration General Hospital, 546 42 Thessaloniki, Greece; marina@med.auth.gr

**Keywords:** hemophilia, female, carrier, bleeding tendency

## Abstract

Hemophilia is no longer regarded solely as a male disorder; female carriers are increasingly attracting the attention of the scientific community due to the common, albeit overlooked, bleeding tendency associated with their condition. Moreover, women can, in rare cases, experience disease severity comparable to that of hemophilic males. The overall bleeding phenotype in females is complicated by gynecological and obstetric bleedings, which do not always correlate directly to their factor levels. As a patient population, they remain significantly underdiagnosed and inadequately treated due to a range of contributing factors. In genetic terms, a carrier exhibits no clinical or laboratory pathology. In that context, the term “hemophilia carrier” has historically led to the presumption of normal hemostasis, thereby impeding the provision of appropriate healthcare services to females in need. Females are not represented in clinical trials and even hemophilia centers lack sufficient experience and appropriate infrastructure to effectively manage and monitor females with hemophilia. Emerging data regarding the needs of this population should be enriched by larger studies, and previously established management practices should be reevaluated, potentially incorporating newer therapeutic options.

## 1. Introduction

Hemophilia has, traditionally, been associated with the male gender due to the X-linked inheritance pattern. Females have been considered as only carriers of abnormal alleles and, thus, expected to maintain normal hemostasis [1]. As early as the beginning of the 1800s, daughters of hemophiliacs were known to transmit the disease and, even though they were presumed not to be clinically affected themselves, were urged not to marry so as not to give birth to an affected male. Even in those early times it had, indeed, been observed that some female carriers experienced prolonged menstruation; however, it was not considered of relevance [2]. This observation has been reinforced in recent years, with additional evidence highlighting a formerly overlooked bleeding tendency in female carriers [3].

In the rare case of females with two abnormal alleles, they are, of course, expected to present the same phenotype as affected males. However, with no difference existing between genders regarding age of first joint bleed, hemophilia diagnosis is later established in females than males. Delayed diagnosis and, therefore, therapy puts females at an increased bleeding risk [4].

Heterozygotes are usually unaffected, as they are protected by the presence of a normal gene on the second X chromosome. Until recent years, the bleeding phenotype in heterozygotes was a subject that has been under-evaluated and under-reported, with sparse literature focusing on adults. To that end, the number of women included in registries is limited, although every hemophilic male corresponds to up to five potential heterozygous females and 1.56 true somatic carriers [5,6]. Due to the bleeding manifestations overlapping with those of females without a bleeding disorder, heterozygotes rarely look for special care and treatment. The limited representation of women and girls in hemophilia surveillance datasets limits the potential to accurately characterize their phenotype and respective needs [3].

The present review aims to raise physician awareness regarding the disease burden among females with hemophilia, highlighting differences in diagnosis and management practices between patients of different sexes, in a disorder that has experienced remarkable therapeutic advances over the past decade.

## 2. Pathophysiology and Terminology

Although rare, women may present with a severe phenotype, either as homozygotes or compound heterozygotes, carrying one mutation from an affected father and one from a carrier mother, or as hemizygous, carrying a hemophilia gene in the presence of a structural or numerical aberration of the other X chromosome [5].

Classic heterozygotes carrying one affected gene may present with a wide range of factor levels. Besides the mutation itself, the factor level variability has been attributed to a possible inactivation of the X chromosome, which leads to cells containing only a single active X chromosome [5]. Random X inactivation is a process ensuring that X-chromosomal genes are expressed equally in both males and females [7]. Preferential X chromosome inactivation over another could influence the phenotype of X-linked disorders in female heterozygotes [8,9,10]. According to one report, skewed X inactivation resulted in female monozygotic twins presenting with clinical discordancy, with one baby being health and one suffering from severe hemophilia A [11]. Interestingly, a Norwegian study in 46 hemophilia A and B carriers found no correlation between X chromosome inactivation patterns and factor plasma concentration [12].

Heterozygotes with factor levels below 0.40 IU/mL should, undoubtedly, be considered as hemophilia patients [3,13]. However, it is widely believed that, like in the case of von Willebrand disease, bleeding susceptibility in hemophilia carriers may start from the low end of the laboratory normal range [14]. In an effort to establish a uniform terminology, so as to improve the recognition and management of affected females, the Scientific and Standardization Committee (SSC) of the International Society on Thrombosis and Haemostasis (ISTH) identified five categories based on bleeding history and baseline factor levels: females with mild, moderate or severe hemophilia (FVIII or FIX > 0.05–0.40 IU/mL, 0.01–0.05 IU/mL or <0.01 IU/mL, respectively), as well as symptomatic and asymptomatic hemophilia carriers (FVIII or FIX > 0.40 IU/mL with or without bleeding symptoms, respectively) [3]. Given that the normal range of FVIII and FIX levels is 0.50–1.50 IU/mL, this categorization addresses the gap regarding the characterization of females with factor levels 0.40–0.50 IU/mL [15]. Along this line, a revised nomenclature has proposed a factor threshold of 0.50 IU/mL as criterion for the classification of a person as a hemophilia patient [16].

## 3. Epidemiology

As commonly reported in other bleeding disorders, factor deficiency in women is underestimated and the overall prevalence unknown [17,18]. Even in cases of a known family history, female carrier status tends to be ignored, and a genetic test is only offered for purposes of prenatal counseling, due to the possibility of pregnancy with an affected male fetus [13]. It was not until 2012 that the World Federation of Hemophilia formally recognized the fact that women could be diagnosed with hemophilia [19].

It is estimated that one third of carriers has a low or mildly reduced factor level (<0.60 IU/mL) [17], while almost 25% of the heterozygotes have factor levels below the haemostatic range [5]. Furthermore, the factor VIII level in heterozygotes demonstrates fluctuation over the course of life—with the highest values at very young and very old ages and nadir occuring between the 25th and 30th year of life [20]. Therefore, a single FVIII measurement during early adulthood might not be representative of the levels observed in previous years, unlike FIX levels that remain stable throughout life after infancy [21].

A report on the Annual Global Survey, published by the World Federation of Hemophilia in 2024, recognized 10,945 female hemophilia patients in 120 countries. The vast majority of identified patients were in high- and upper-middle-income countries and had mild hemophilia with both hemophilia A and B [22].

Previously, Miller et al. analyzed data from the Community Counts Public Health Surveillance project during the period 2012–2020 in order to characterize female patients with hemophilia [21]. Among patients with hemophilia receiving care in hemophilia centers in the USA, 1667 females with factor levels <0.40 IU/mL were identified. One in five patients with mild hemophilia was female, while the severe or moderate phenotype was uncommon, as expected. Interestingly, female patients had fewer Hemophilia Treatment Center visits compared to males across all severity categories.

Recently, Krumb et al. reported on systematic screening in an effort to identify female carriers among 236 families of patients with hemophilia followed at a single center at the Cliniques universitaires Saint-Luc in Brussels [23]. In total, 454 females were identified as hemophilia A or B carriers, while 23% and 29.5% of those with known factor VIII and IX levels, respectively, presented a factor deficiency (<0.40 IU/dL). It is noteworthy that only two hemophilia carriers were regularly followed

Similarly, prompted by a woman with moderate hemophilia, Wang et al. identified, in a family with consanguineous marriage, one female with hemophilia and 18 carriers using pedigree tracking and genetic testing [24].

## 4. Clinical Phenotype—Obstetric and Gynecological Issues

The symptoms of females with hemophilia are the same as in males and are dependent on the residual factor level [1]. Consequently, females could present with a full clinical expression of hemophilia, including hemarthosis [3].

However, compared to men, women face additional hemostatic challenges throughout their lives. The female-specific bleeding phenotype is often complicated by gynaecologic and obstetrical bleeding [5,25]. Therefore, data regarding the health-related quality of life reported in hemophilic males cannot be extrapolated to females with hemophilia. To that end, reports on women with bleeding disorders are now highlighting that their disease status adversely affects their daily life and overall well-being [26].

In the past, studies on carriers of hemophilia mainly focused on genetic counseling, as well as the psychological burden of passing on the disease to future generations [27,28,29]. According to current emerging data, hemophilia heterozygosity seems to have an additional clinical impact on the reported quality of life. A cross-sectional questionnaire completion study demonstrated significantly lower scores in the domains of “Pain” and “General Health” in 42 hemophilia carriers compared to controls [30]. Even though there is a certain response bias in questionnaire-based research, adding a comparison group strengthens study validity, allowing for causal inferences to be drawn [31].

Compared to healthy women, heterozygotes show higher bleeding scores with regard to cutaneous, minor wound, oral cavity, menstrual, postsurgical and postpartum bleeding. It is of particular interest that excessive bleeding is reported even in carriers with normal factor levels [5].

With regard to pregnancy, female carriers increase their clotting factor levels—mainly FVIII and to a much lower extent FIX—similarly to healthy pregnant individuals, although these levels do not, typically, reach those observed in normal pregnancies. The factor levels start to decrease from the third postpartum day, returning to the woman’s baseline within one week, thereby increasing the risk for bleeding. Postpartum hemorrhage (PPH) occurs at a much higher rate in females with hemophilia compared to the general population [32,33,34]. More specifically, according to a recent systematic review and meta-analysis of 81 studies that included 42,709,185 women, the pooled prevalence of PPH using objective blood loss assessment methods was 12.6% and 8.2% after vaginal delivery and cesarean section, respectively [35]. In the case of hemophilia carriers, however, a systematic review reported on PPH in 55 (63%) out of 88 deliveries, with lower, albeit, significant incidence even after the application of prophylactic interventions (43.6% with vs. 77.1% without prophylactic treatment, respectively) [32]. Moreover, a multicenter retrospective Dutch cohort study in 114 pregnancies of 93 hemophilia carriers demonstrated PPH in 35 (30.7%) cases, over a 10-year time period [36], while another single-center English retrospective study of 65 pregnancies in hemophilia carriers revealed a PPH incidence of 19% [33].

Almost two decades ago, Plug et al. conducted a postal survey of women that had been tested for hemophilia carriership in the Netherlands using a questionnaire that included, among others, items on history of bleeding events [37]. A total of 546 women responded, including 274 carriers of hemophilia A or B, with the survey concluding that carriers bled more compared to noncarriers, especially after medical interventions. Increased bleeding tendency was not only observed in factor levels indicative of mild hemophilia, but also in factor levels of 0.41–0.60 IU/mL [37].

Similarly, in a multinational, prospective, observational, cross-sectional study performed by members of the Global Emerging Hemostasis Panel, James et al. reported on 168 hemophilia carriers that experienced more bleeding episodes, not only in comparison with the normal female population, but also with von Willebrand type 1 disease patients [38]. Notably, six carriers in the cohort with normal factor levels presented with joint bleeds [38].

An increased bleeding tendency, identified as frequent and recurrent bleeding, i.e., recurrent easy bruising in unusual areas, recurrent nose bleeding, recurrent gum bleeding, prolonged or heavy menstrual bleeding for more than 5 days, prolonged bleeding after giving birth or after surgical procedures, was reported in a single-center, German observational study. The report included 46 hemophilia A carriers and correlated the bleeding tendency to the severity of the disease in the male hemophilic, as well as the causative gene mutation [39]. These findings contradict a prior Austrian study that included 42 hemophilia A carriers, which found no correlation between mutation type and FVIII levels [40].

More recently, in a retrospective, non-interventional, multicenter study, Chaudhury et al. reported increased menstrual bleeding as the referral symptom of 47 hemophilia carriers followed in the USA, with difficult management even with antifibrinolytics [17]. In addition, excessive bleeding was reported during or after delivery, requiring factor concentrate, antifibrinolytics and/or transfusions. Joint bleeds were reported in 19% of the cohort population. Similarly, Paroskie et al. had earlier studied the bleeding phenotype of 44 carriers compared to normal women in a cross-sectional study, and demonstrated more frequent bleeding after dental extraction (72%), heavy menstrual bleeding (67%), hematomas, epistaxis, postsurgical and peripartum bleeding, as well as joint bleeds in 19% of the study population [41].

Despite concerns that hemorrhagic events may be overreported by female carriers, an increasing body of evidence substantiates their existance [42]. Gilbert et al. prospectively studied 30 hemophilia A carriers and reported on joint abnormalities in nine of them, which were either clinically evident with a reduced range of motion and/or radiologically present in the form of haemosiderin deposition according to MRI, indicating previous subclinical joint bleeds [14].

Another multicenter, cross-sectional, Chinese study with 125 obligate hemophilia carriers and healthy controls described increased bleeding scores using the International Society on Thrombosis and Haemostasis Bleeding Assessment Tool (ISTH-BAT), especially after surgical abortion and intrauterine device placement, although bleeding score was not correlated with factor levels [43].

Finally, based on the American Thrombosis & Hemostasis Network dataset (ATHNdataset), in a cohort of 626 pediatric carriers, nearly half fell within the spectrum of mild hemophilia, but only 13.5% presented abnormal bleeding scores. However, among the remaining carriers, 10% were found to have abnormal bleeding scores [44] (Table 1).

It is becoming evident that in female carriers, as in male patients, factor levels alone do not reliably predict bleeding tendency [45]. In that context, bleeding episodes indicate inadequate or absent preventive measures that could (and should) have been applied, especially in the case of invasive procedures.

## 5. Management Strategies

Females with hemophilia remain underreported, even though disease inheritance seems to have an impact on both sexes [18,46]. Recent research demonstrates higher morbidity and mortality rates among both male and female individuals with hemophilia relative to controls, underscoring the need for management optimization, irrespective of disease severity or sex [47].

Hemophilia carriers’ referral in specialized treatment centers is highly recommended. However, the vast majority of pregnant heterozygotes do not have specialist care before (if that) the time of delivery [13]. An online survey of 280 women with bleeding disorders demonstrated that hemophilia carriers were the least likely to be followed in a specialized setting when compared to women with other bleeding disorders or immune thrombocytopenia [48].

It is suggested that affected women and girls could be identified during follow up of their hemophilic male relatives [13]. Active testing of potentially affected individuals in a family is recommended as the key to early diagnosis and subsequent referral. In the absence of guidelines, a variety of practices concerning hemophilia screening have been reported [49]. Given the limitations of genetic testing, i.e., cost and availability, factor levels can be easily assessed during childhood and facilitate carrier identification [50].

The “normalization” of bleeding symptoms within families affected by bleeding disorders could impede the timely identification of females with hemophilia [13]. Bleeding phenotype can be easily characterized using the Bleeding Assessment Tool (BAT) [51], which is considered particularly useful in everyday clinical practice. Reference ranges are defined separately for adult women and children; it is, however, advised that the classification of symptomatic carriers does not solely rely on bleeding score criteria. Evaluation of both laboratory and clinical patient characteristics should be undertaken by experienced health care professionals at hemophilia treatment centers [3].

Females with hemophilia require on-demand and/or prophylactic treatment in dose and frequency that does not differ from that of males with the same disease severity—although women and girls are infrequently represented in clinical trials [52]. Similarly to male patients, an individualized approach depending on bleeding phenotype is recommended [53]. As for inhibitor development and relative management, data in females is limited and only depicted in case reports or small case series [54,55].

Hemophilia carriers exhibiting greater bleeding than males with equivalent factor levels should be assessed for coexisting bleeding disorders, such as von Willebrand disease or congenital platelet defects [53].

Females with hemophilia, as well as carriers, should be educated regarding the expected hemostatic challenges throughout their lives, like the increased risk for heavy bleeding at menarche, perimenopause or childbirth. Menstrual blood loss should be assessed using pictorial bleeding assessment charts, as self-reporting bleeding is often inaccurate. In addition, hemoglobin levels and iron stores should be regularly monitored. It is important to ensure access to care for both female-specific and general bleeding symptoms, while management protocols should be applied [13]. Ideal factor levels to maintain hemostasis during menstruation have not been established. Treatment algorithms include hemostatic medication (such as tranexamic acid, desmopressin or factor concentrate), hormonal agents or a combination of both. It is of importance that when analgesia is required, the use of non-steroidal anti-inflammatory drugs is discouraged, due to their effect on platelet function [56].

As for childbirth, the World Federation of Hemophilia Guidelines for the Management of Hemophilia (3rd Edition) has recommended a minimum maternal factor level threshold of 0.50 IU/mL [53], along with careful planning for delivery in specialized centers [57,58]. Peripartum management includes the administration of factor concentrate, desmopressin and/or antifibrinolytics; however, there is no consensus on the optimal treatment to prevent postpartum hemorrhage and different hematological and gynecological approaches have been reported [32,59].

Unfortunately, even some hemophilia treatment centers (HTCs) appear to be inadequately prepared to accommodate and manage women with hemophilia, lacking proper multidisciplinary clinics with participating hematologists, gynecologists, nurses and psychologists, in addition to prenatal diagnostics and management protocols for menorrhagia [60,61]. A systematic effort has been reported in the United States over the last decade, aiming at providing special care for females in HTCs by organizing interdisciplinary clinics. However, the number of female patients with hemophilia treated within HTCs remains much lower than expected based on disease epidemiology [46]. The National Hemophilia Foundation emphasized the need for improvements in comprehensive HTC care to increase equitable access for women with hemophilia [62].

## 6. Gaps in Evidence and Future Directions

Cumulative evidence highlights the importance of identifying and closely monitoring females with hemophilia to ensure proper preparation for hemostatic challenges and optimal clinical management. Pediatric centers could play a key role in the early identification of hemophilia carriers and females with hemophilia by offering family screening during routine visits of male patients with hemophilia.

Furthermore, an evidence-based approach to care for a range of clinical scenarios prerequisites the inclusion of females with hemophilia in research and clinical trials [63]. Despite significant advances in therapeutic options in recent years, females have not equally benefited from this progress compared to males with hemophilia [64].

For hemophilia carriers who experience bleeding despite normal clotting factor levels, the underlying pathophysiology of impaired hemostasis should be investigated in order to improve diagnostic accuracy and therapeutic strategies [65]. An increased age-related risk for abnormal bleeding, simultaneously with rising factor levels, has been recently described as the “carrier paradox”. A discrepancy in mean factor levels between girls and adult women who are carriers has been documented (approximately 47–48 IU/dL vs. 65–70 IU/dL, respectively), suggesting that factor levels tend to increase with age. However, bleeding scores appear to increase as well, highlighting a paradoxical discordance between laboratory measurements and clinical phenotype [66]. Apart from potential biological modifiers that may contribute to bleeding tendency but remain insufficiently characterized, methodological and analytical pitfalls in laboratory assessment may also play a contributing role. Pre-analytical variables, assay variability, and differences between one-stage and chromogenic factor assays may result in misclassification, potentially categorizing a carrier within the normal range despite a clinically relevant bleeding phenotype. The use of chromogenic assays for factor level measurements, in contrast to the commonly used one-stage assay, may reclassify individuals previously considered hemophilia carriers into the category of mild hemophilia. Although chromogenic assays are reported to be less frequently used in the diagnostic evaluation of women with hemophilia—primarily due to cost and limited availability—there is likely a need to incorporate both assay methodologies into bleeding risk stratification, particularly in the perioperative setting [67,68]. Moreover, the safety threshold of 0.50 IU/mL factor levels set for delivery may not adequately protect pregnant women, particularly in view of the physiological fluctuations in FVIII and FIX levels and the potential risk of bleeding if levels fall below the protective range at the time of childbirth.

In the context of an overall limited number of identified female patients worldwide, an even smaller number of identified pediatric patients exists. For example, only 27 girls were included in reports across 34 pediatric HTCs in Europe, based on the annual 2024 report of PedNet (European Pediatric Network for Hemophilia management) [69]. Given the rarity of the patient group, insufficient clinical experience among many health care providers is to be expected. Although the Women and Girls with Bleeding Disorders Clinic of Excellence Model is becoming the standard of care in the United States, with more than 70 designated interdisciplinary clinics [70], numerous HTCs remain insufficiently prepared and inadequately structured to effectively respond to the unique challenges encountered by women with hemophilia. This gap was highlighted in a previous electronic survey conducted across 59 HTCs in Europe [60].

Studies encompassing larger cohorts of affected females, along with comprehensive reevaluations of clinical guidelines pertaining to the management of this population, is imperative.

## Figures and Tables

**Table 1:** Published studies regarding clinical phenotype in hemophilia carriers.

Reference	Study Design	N (Carriers)	Key Findings
Plug et al. (2006) [37]	Postal survey	274	▪ Increased bleeding scores vs. noncarriers ▪ Bleeding events even at FVIII > 0.40 IU/mL
James et al. (2016) [38]	Prospective cross-sectional	168	▪ Increased bleeding scores vs. healthy women and women with von Willebrand disease type 1 ▪ Joint bleeds in 6 carriers with normal factor levels
Olsson et al. (2014) [45]	Observational	46	▪ Bleeding phenotype correlated with mutation type
Ay, et al. (2010) [40]	Observational	42	▪ No mutation–FVIII levels correlation
Chaudhury et al. (2021) [17]	Retrospective multicenter	47	▪ Referral symptom was heavy menstrual bleeding ▪ Joint bleeds in 19% of cohort population
Paroskie et al. (2015) [41]	Cross-sectional	44	▪ More frequent bleeding after dental extraction and heavy menstrual bleeding ▪ Joint bleeds in 19% of cohort population
Gilbert et al. (2014) [14]	Prospective	30	▪ 9/30 joint abnormalities with reduced range of motion or MRI hemosiderin deposition
Li et al. (2020) [43]	Multicenter cross-sectional	125	▪ Increased bleeding score vs. healthy women ▪ No correlation between bleeding score and factor levels
Puetz et al. (2023) [44]	Registry-based	626 (pediatric)	▪ Mild hemophilia in 50% of study cohort ▪ Increased bleeding scores in 13.5% of females with mild hemophilia and in 10% of carriers with factor levels in normal range

## Data Availability

No new data were created or analyzed in this study. Data sharing is not applicable to this article.
